# Closed-system automated microdistillation of small-volume water samples for tritium and stable isotope analysis

**DOI:** 10.1016/j.mex.2026.103958

**Published:** 2026-05-15

**Authors:** Daisy Kazeneza Butera, Leonard I. Wassenaar, Anthony Lapp, Paul Middlestead, Oliver Warr

**Affiliations:** University of Ottawa, 25 Templeton St, Ottawa, ON K1N 7P6, Canada

**Keywords:** Tritium analysis, Microdistillation, Water sample preparation, Electrolytic enrichment, Closed-system distillation, Stable isotope preservation

## Abstract

Preparation of small-volume water samples for low-level tritium (³H) analysis commonly requires microdistillation to remove electrolytes and dissolved salts following electrolytic enrichment. This step must efficiently purify the sample, achieve quantitative water recovery, preserve hydrogen and oxygen isotope ratios, and avoid sample cross-contamination. Existing distillation approaches are often time and labor-intensive, require continuous operator supervision, and/or are poorly suited to batch processing of small volumes. This article documents a closed-system, automated microdistillation method for purifying small-volume (<20 mL) water samples prior to tritium and stable isotope analysis. The method is based on paired, chemically inert vessels connected in a sealed configuration and operated under controlled heating conditions. Validation data are presented demonstrating efficient gravimetric recovery of the water sample, quantitative removal of dissolved salts, accurate preservation of hydrogen and oxygen isotope ratios across a wide range of salinities and isotopic compositions, and negligible long-term memory effects for deuterium- and tritium-enriched samples. The method is intended for routine use in tritium laboratories processing electrolytically enriched samples and other stable isotope laboratories requiring reproducible microdistillation.

• Automated closed-system microdistillation of small-volume water samples.
• Batch processing under controlled thermal conditions.
• Validation of recovery, isotopic integrity, and carryover performance.

## Graphical abstract

**Figure:**
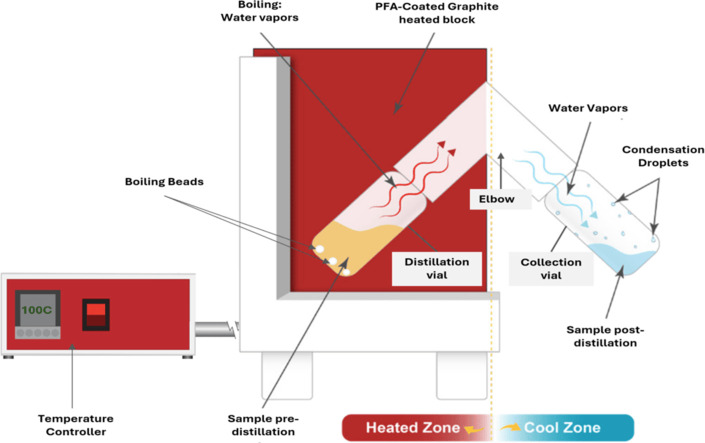

## Specifications table

**Table:** 

Subject area	Environmental Science
More specific subject area	Environmental radioactivity; isotope hydrology; water sample preparation.
Name of your method	Closed-system automated microdistillation of small-volume water samples for tritium and stable isotope analysis.
Resource availability	EvapoClean automated microdistillation system (Analab–Elemental Scientific, www.analab.fr).

## Background

Accurate determination of low-level tritium (³H) in environmental waters critically depends on sample preparation. When activities are sufficiently high, purified small-volume water samples (8–12 mL) can be directly measured via liquid scintillation counting (LSC). However, in modern precipitation, groundwater, and surface waters, tritium activities are commonly near natural background levels, requiring an additional step of electrolytic enrichment of larger water volumes prior to LSC analysis [1,2]. This electrolytic ^3^H-enrichment procedure reduces initial sample volumes of 250–2000 mL to final sample volumes of approximately 8–12 mL in order to concentrate tritium by factors of 15–100x to achieve LSC detectable levels [3]. This electrolytic enrichment process produces highly alkaline, conductive concentrates that must be neutralized and purified before LSC analysis can proceed [4].

Residual electrolytes in enriched samples or natural dissolved salts in environmental samples must be removed as they interfere with LSC measurements by quenching the scintillation cocktail and reducing counting efficiency. To address this, microdistillation of small-volume samples is a required preparative step to ensure purified samples with no residual electrolytes [5]. In addition to electrolyte removal, quantitative confirmation of water recovery during microdistillation in a closed system is required to ensure that the original sample hydrogen and oxygen isotope ratios and tritium are preserved. Partial or incomplete distillation may preferentially transfer lighter isotopologues to the vapor phase or cause a loss of ^3^H, altering the H and O isotopic composition of the recovered water sample. As deuterium (²H) is enriched concurrently with ^3^H during electrolytic enrichment, it is reliably used to determine tritium enrichment factors. Preservation of both rare and abundant hydrogen isotopes during purification is therefore critical to ensure accurate calculations of environmental tritium concentrations [5].

Water purification has long been addressed in isotope hydrology, using classical boiling, azeotropic, or vacuum distillation approaches [6], typically with large-volume (e.g., > 250 mL) glassware and cooling-coil or cryogenic condensation-based recovery [7]. While these classical methods produce high-purity water, they are time and labour-intensive, require continuous supervision, may involve open-system condensation, and require subsequent cleaning of glassware to prevent carryover between distillations. As tritium and stable isotope applications have expanded to include electrolytically enriched samples, pore waters, laboratory tracer studies, and precipitation monitoring, there is an increasing need for automated, closed-system microdistillation approaches [8,9].

Despite the widespread use of classical distillation approaches, these methods are not optimized for closed-system processing of small-volume (<20 mL) samples typical of electrolytically enriched tritium workflows. Most small-volume methods focus on soil or plant water extractions [10,11]; however, they are poorly suited to batch processing, may require continuous operator supervision, and can introduce risks of evaporative loss, atmospheric exchange, or cross-contamination. There is therefore a need for a reproducible, automated microdistillation procedure that enables quantitative recovery, preserves isotopic integrity, and supports routine high-throughput laboratory operation.

Tritium laboratories typically process batches of water samples for routine environmental monitoring, hydrological investigations, and nuclear safety programs. Under these operational conditions, sample preparation workflows must accommodate repeated distillations, saline or alkaline matrices, and consistent purity outcomes across batches. To streamline this, automation is used in analytical laboratories to help standardize preparative steps and reduce operator involvement and error.

This paper documents a closed-system, automated microdistillation procedure for purifying small-volume (< 20 mL) water samples. The method is described in detail to enable replication and is accompanied by validation data confirming accurate and reproducible water recovery, preservation of hydrogen (^2^H and ^3^H) and oxygen isotope ratios, sample neutralization and purification, and assessment of ^2^H and ^3^H sample memory effects. The approach is intended for routine automated preparation of electrolytically enriched tritium samples and other small-volume water matrices requiring microdistillation in tritium and stable-isotope hydrology laboratories.

## Method details

### Equipment and materials

**Fig. 1:**
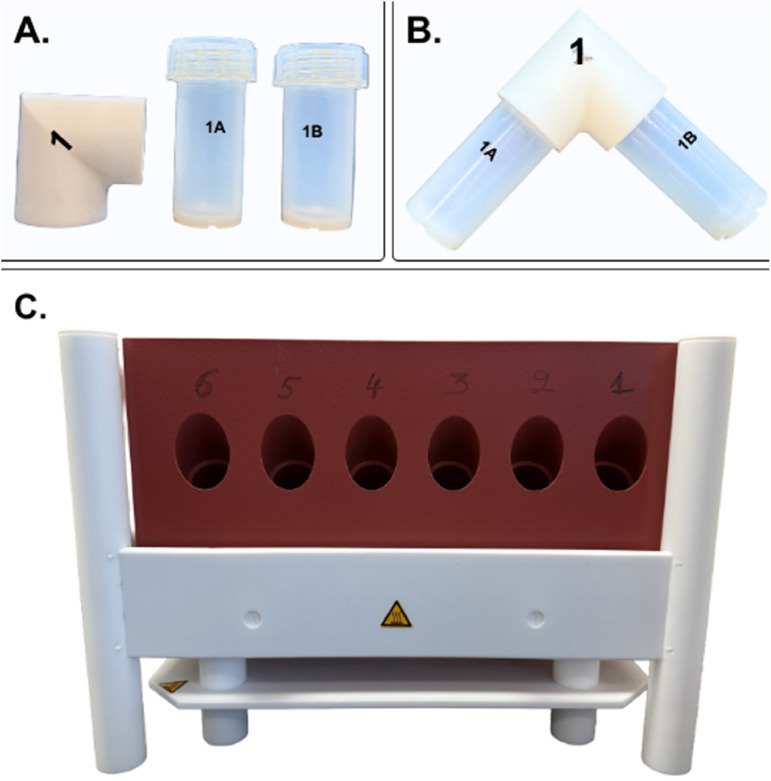
(A) Individual capped EvapoClean PFA vials: distillation vial (1A), collection vial (1B), and PTFE elbow. (B) Assembled distillation setup: distillation vial and collection vial connected to both ends of the elbow. (C) Front view of the heating block showing six empty purification ports (manually labeled 1–6).

Automated water sample microdistillation was performed using a commercial system (EvapoClean Model EV-12.25–1V-15A; 110 V) with an external controller and timer (Model RNP-1V-15A-Tl; 110 V) from Analab-Elemental Science (www.analab.fr). The system required CSA (Canadian Standards Association) certification before use. The system consists of a PFA-coated graphite heating block with sample ports and an external temperature controller with programmable timings. The system used was a 12-port version supporting 25 mL distillation vessels. Ports on the heating block were numbered 1–12 to correspond to each set of vessels (Fig. 1). Fig. 1 shows only the front view of the EvapoClean heating block, displaying ports 1–6; the back view is a mirror image of the front view and contains the remaining ports 7–12.

**Fig. 2:**
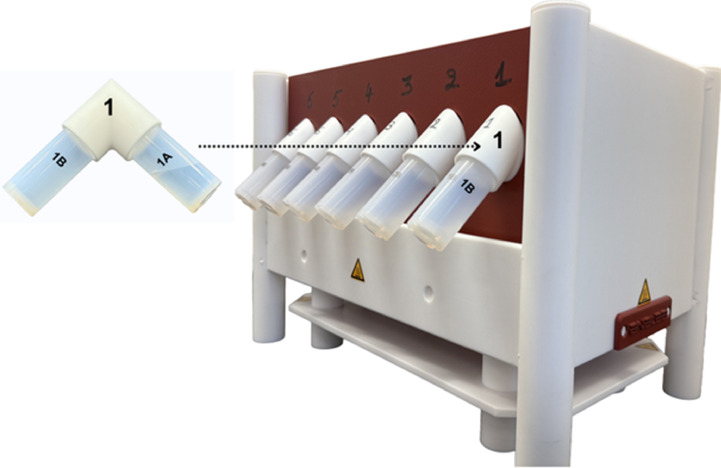
Front view of the heating block with assembled vials prior to distillation.

Each 25 mL distillation vessel comprises two chemically inert PFA GL 32 threaded vials: a hot distillation-side vial and a room-temperature collection-side vial. Two PFA caps were used to tightly seal and store samples before and after distillation. The vials were connected by a threaded fluoropolymer elbow to form a closed-system angled distillation pathway when tightly sealed. The distillation-side vial containing the water sample was placed inside the purification port, while the collection-side vial was held outside the heating block at ambient laboratory temperature (see graphical abstract and Fig. 2). This closed-system configuration eliminated exposure to the laboratory air moisture and evaporative loss during operation. The system was operated on a laboratory benchtop. All vials and connecting elbows were individually numbered on their caps and vial bodies to ensure matched sets were used continuously.

A manufacturer-supplied controller was connected to the heating block to regulate temperature and timing. The controller allowed manual temperature selection and timed operation for unattended distillations. For all validation microdistillation experiments described in this study, the heating block was set to 100 °C (± 0.1 °C), except for the combined sample neutralization-and-distillation validation test, which was set to 155 °C. All experiments were run for 18 h; this duration was selected in our laboratory to allow overnight distillation and accommodate staff schedules; shorter distillation times are feasible but would require further validation. Eighteen hours represents a conservative duration ensuring complete transfer under all tested salinity and alkalinity conditions; shorter times (<12 h) may be feasible but were not evaluated here. Allow approximately 1 hour for the heating block to reach the target operating temperature. To ensure thermal stability, the unit should be operated in temperature-controlled environments.

Three solid glass boiling beads (3 mm diameter; Fisher Scientific, 11–312A) were added to the distillation vials to promote controlled boiling and to prevent liquid or salt spray carryover to the collection vial during distillation. All vials, caps, elbows, and boiling chips were handled with powder-free laboratory gloves to minimize external contamination.

Sample masses were determined using a Mettler Toledo ME4002E high-precision laboratory balance with a readability of ± 0.01 g.

The electrical conductivity of the sample solutions was measured before and after distillation using a calibrated laboratory conductivity probe (Mettler Toledo, Seven Excellence™ Conductivity Meter S700) suitable for both high-salinity solutions and low-conductivity waters. For downstream analyses, acceptable electrical conductivity thresholds are < 60 µS cm⁻¹ for liquid scintillation counting and < 300 µS cm⁻¹ for stable-isotope analysis [12].

Indicator strips were used to assess pH before and after the combined neutralization and distillation tests.

Reagents included: deionized laboratory water, tritium-free water, reagent-grade sodium chloride (NaCl), commercial aquarium sea salt, deuterated water (D₂O, ≥99.9 %), lead chloride (PbCl₂) for neutralisation of alkaline samples resulting from electrolytic enrichment, and a certified tritium standard (NIST SRM 4926E). All reagents were used as received unless otherwise stated. Toxic chemicals and radioisotopes were handled and disposed of in accordance with the University of Ottawa Health and Safety guidelines.

### Distillation procedure

This procedure describes microdistillation of small-volume water samples using a closed-system, two-vial EvapoClean vessel kit (distillation vial + collection vial connected by a fluoropolymer elbow, caps). The procedure is written for twelve 25 mL vessels and a heating block operated at 100 or 155 °C. Throughout this study, water quantities are defined gravimetrically (1 g ≈ 1 mL at laboratory temperature).

#### Sample preparation and pre-weighing

1. Inspect all EvapoClean components. Verify that the distillation vial, collection vial, caps, and connecting elbow are clean, dry, and free of visible residues or damage. Ensure that numbered vials and caps are correctly matched. Add three clean glass boiling beads to each distillation vial.
2. Weigh all components with caps installed and record the mass of each vial. This step is required as vials are capped immediately after distillation to minimize evaporative loss, and post-distillation weighing must be comparable. The distillation vial is weighed with the glass beads in place.

#### Sample loading and vessel assembly

1. Add water samples by mass, not volume. Weigh 12.0 g of sample (or the application-specific target mass, up to a maximum of 20.0 g) into the pre-weighed distillation vial containing the glass beads. Record the final pre-distillation mass. For the samples requiring neutralization, weigh 4 g of PbCl_2_ in a separate disposable vial and add it to the distillation vial after the enriched sample has been added.
2. Cap the distillation vial immediately and firmly to prevent evaporation while preparing other samples.
3. When ready to distill, assemble each vessel. Uncap the distillation vial, hold it vertically to avoid spillage, and screw into the fluoropolymer elbow. Then connect the collection vial to close the system.
4. Tighten both vial-to-elbow connections firmly to ensure a closed system pathway to prevent evaporative leakage or loss. Avoid overtightening to prevent thread stripping, or deformation. Ensure the distillation vial containing the sample always remains upright to prevent cross contamination with the collection vial.

#### EvapoClean distillation runs

1. Load the samples by placing each numbered assembled vessel into the corresponding ports of the EvapoClean heating block. The distillation vial containing the sample, PbCl2 (if applicable) and boiling beads is placed inside the port, and the collection vial remains outside the heating block at ambient temperature.
2. Set the operating temperature at 100 °C or 155 °C (for samples without or with PbCl₂, respectively), and program the controller for an 18-hour overnight distillation to allow unattended operation.
3. Do not open or remove sample vials during the distillation. Maintain the closed-system configuration throughout the run.
4. At the end of the 18-hour distillation period (e.g. the following morning), switch off the heating block before removing the sample vials. The vials should be removed while the heating block remains hot to prevent vapor backflow, which could cause condensed water in the collection vial to migrate back into the distillation vial.

#### Post-distillation handling and weighing

1. After completion, remove the sample vessel assemblies (caution: still hot) from the block. Inspect for recovery or leakage. The vial bodies are translucent, and the distilled water sample should be visible only in the collection vial. Ensure that no spillage from the collection vial to the distillation vial occurs during handling.
2. Unscrew the collection vial and immediately cap it to prevent evaporative water loss.
3. Disassemble the connection elbow and distillation vial, cap the distillation vial and set the parts aside for cleaning. The distillation-side components are hot; appropriate thermal protection (e.g., heat-resistant gloves) should be used during handling.
4. Repeat steps 2 and 3 for all remaining samples.
5. Weigh the capped collection vials and record the post-distillation mass of the recovered water sample distillate. It is important to note that, in this study, deionized water was used to estimate gravimetric recovery as part of the performance validation of the EvapoClean system. During routine operation, pre- and post-distillation masses are still recorded as good laboratory practice to monitor approximate water recovery; however, they are not used for quantitative gravimetric calculations because expected recoveries cannot be precisely defined due to variable sample salt content.
6. If required, calculate mass water recovery using the pre- and post-distillation masses as defined in the Statistical evaluation section.

#### Transfer and sample storage

1. Measure the electrical conductivity of the distillate. Open the collection vial and, using a clean, dry, calibrated conductivity probe, record the electrical conductivity of the sample. For ^3^H analysis, acceptable conductivity is < 60 µS cm⁻¹; for stable isotope analysis, values up to <300 µS cm⁻¹ are acceptable. High values may indicate a failed distillation or carryover of salt spray from the distillation vial. Samples exceeding the conductivity threshold for the intended isotope analysis must be reprocessed using the above protocol. Clean and dry the conductivity probe between samples.
2. For the combined neutralization-distillation test, also measure the sample pH. While the vial remains open following the conductivity measurement, use a pipette to place a drop of the sample onto pH indicator paper and record the pH. Samples with a neutral pH of approximately 7 are acceptable for LSC.
3. Always keep the sample vial firmly capped except during required subsampling to prevent evaporative loss or exposure to atmospheric moisture.
4. For subsampling for stable-isotope (e.g. 1 mL) and tritium (e.g. 8 mL) assays, open the collection vial and, using a new disposable pipette, transfer the appropriate amount of water to the corresponding stable-isotope or tritium LSC sample vials. Label each vial with the sample ID, date, and distillation port position (if relevant) and store subsamples capped at laboratory temperature (or as required by downstream isotope analysis).
5. Excess distillate sample can be discarded or stored temporarily in 2 mL glass GC vials for possible repeat analysis.

#### Between-run cleaning

Once all subsamples have been collected, proceed to cleaning.

1. Recover the boiling beads from the distillation vials. For processed samples containing salt residue, add tap water to the distillation vial to dissolve the salt and rinse the contents through a strainer to recover the boiling beads. Collect the salt residue in a designated hazardous waste container for salt waste.
2. Soak all empty vials, caps, beads, and connecting elbows in a soap bath (laboratory detergent) for 30 min, then clean each item thoroughly with a sponge.
3. Rinse all components and soak them in deionized water for > 1 hour, then dry overnight in an oven at 100 °C.
4. After drying, inspect all components for cleanliness. The translucent vials should be free of any visible residue, which would otherwise appear as dried white patches on the interior surfaces.
5. Store cleaned vials, elbows, and boiling beads dry in a drawer until reuse. *Note: Using two sets of vials can improve sample throughput, as one set can be used while the other is being cleaned*.

#### PbCl_2_- specific cleaning

Cleaning of PbCl₂ post-distillation residues requires additional precautions due to lead's toxicity.

1. Work in a fume hood. Discard all solid Pb residue remaining in the distillation vial. Add deionized water to the vial to dissolve the paste-like Pb residue and discard the solution into a liquid waste container designated exclusively for lead. Boiling beads are not recovered during this step and are discarded with the Pb waste. Rinse the vial with at least two additional aliquots of deionized water, discarding each rinse into the Pb waste container. Repeat as necessary until no visible solid particles remain. Do not place the distillation vial into the detergent bath if any visible solid residue remains.
2. Once all visible solid residue has been removed, proceed with the cleaning steps 2 - 5 as described in the cleaning section above.

## Method validation

Validation experiments were designed to independently assess water recovery, salt removal, isotopic integrity, and memory effects under worst-case salinity, alkalinity, and isotopic enrichment conditions.

Stable isotope (δ^2^H, δ^18^O) compositions of pre- and post-distilled samples were measured in the Jan Veizer Stable Isotope Laboratory at the University of Ottawa by laser spectroscopy. The natural abundance samples were analyzed on a Picarro water isotope analyzer (L2130-i), whereas the deuterium-enriched samples were analyzed using the Liquid Water Isotope Water Analyzer (LIWA-45EP) by Los Gatos Research (LGR) [13]. All results are reported on the VSMOW scale.

Tritium measurements were performed in the André E. Lalonde National Facility for Accelerator Mass Spectrometry (AEL-AMS) by liquid scintillation counting (LSC) using a HIDEX Ultra-Low-Level Analyzer (ULLA). Tritium activities (reported in Bq L⁻¹) of distilled test water samples were determined using established techniques (8 mL distilled water sample + 12 mL Ultima Gold scintillation cocktail) using a 500-minute counting time.

### Sample types

Seven categories of water samples were prepared and processed using the distillation procedure described in this article. These sample types were selected to represent various water sample matrices commonly encountered in tritium laboratories and stable isotope hydrology applications, including low-ionic-strength waters, saline and alkaline solutions, isotopically enriched waters, and tritium-bearing water samples.

#### Sample type 1: deionized water

Deionized water (conductivity 0.8 µS cm⁻¹) of known stable isotopic composition was used as the reference matrix for gravimetric recovery and isotope preservation after distillation. This water contained no intentionally added dissolved salts or isotopic tracers and served as a low-conductivity baseline for assessing quantitative water recovery during microdistillation. Aliquots were prepared directly from a laboratory deionized water supply. Aliquots of deionized water used in the tests were analyzed for their initial δ^2^H and δ^18^O compositions and used in subsequent salt-solution tests.

#### Sample type 2: sodium chloride waters

A series of sodium chloride (NaCl) solutions was prepared by dissolving reagent-grade NaCl in the deionized water of known stable isotope composition. Solutions were prepared at 5, 15, 35, 70, and 100 g L⁻¹ to span a wide range of ionic strengths encountered in nature or the laboratory: groundwaters, brackish waters, seawater, concentrated brines, and electrolytically enriched tritium samples. Each concentration was subdivided into replicate aliquots prior to distillation.

#### Sample type 3: sea-salt waters

Sea-salt solutions were prepared by dissolving commercially available aquarium salt in deionized water of known stable isotopic composition at the same concentrations used for the NaCl solutions (5, 15, 35, 70, and 100 g L⁻¹). These solutions represent chemically complex matrices analogous to those found in nature that contain multiple dissolved ions, including monovalent and divalent cations. Replicate aliquots were prepared for each concentration prior to distillation.

#### Sample type 4: deuterium-enriched waters

Deuterium-enriched water samples were prepared by gravimetrically mixing deuterated water (D₂O, ≥99.9 %) with isotopically characterized deionized water to produce solutions with elevated δ²H values to test for recovery and carryover. Target compositions were approximately 10,000 ‰ and 20,000 ‰ (VSMOW), representing electrolytically enriched levels commonly encountered in 250–500 mL electrolytically enriched tritium samples and in laboratory tracer applications. Prepared solutions were homogenized prior to aliquoting and distillation.

#### Sample type 5 & 6: tritium-spiked and tritium-free water

Tritium-bearing samples were prepared by adding a certified tritium standard reference material (NIST SRM 4926E) to deionized water to produce a homogeneous tritium-spiked solution (SPK-2) with a target activity of 35.70 Bq L⁻¹ (300 TU) suitable for direct counting by LSC. This solution was used to evaluate quantitative tritium recovery and potential sample carryover during distillation. Additional tritium-free water samples were processed in each vial following distillation of the spiked solution to assess the absence of detectable ^3^H carryover/cross-contamination between sequential distillations.

#### Sample type 7: enriched tritium samples - combined neutralization and distillation

Electrolytic enrichment of tritium produces highly alkaline samples (typically pH > 13) because sodium peroxide is used as the electrolyte, hydroxide ions are generated in the sample. Before liquid scintillation counting, to avoid a corrosive sample and chemical quenching when the scintillation cocktail is added, these enriched solutions must be neutralized to approximately pH 7 and subsequently distilled. Neutralization must be achieved without introducing any external hydrogen, which would otherwise contaminate the sample's original tritium composition.

Traditionally, neutralization of enriched tritium samples is accomplished either by dissolving and heating solid lead (II) chloride (PbCl₂) directly into the alkaline solution or by bubbling carbon dioxide gas through the solution before distilling the sample in a glass apparatus. In this study and proposed methodology, PbCl_2_ neutralization was used in combination with the EvapoClean distillation.

For this test, aliquots of electrolytically enriched sample (approximately 12 mL) were loaded into the distillation vials with lead chloride added in stoichiometric excess relative to hydroxide (ca. 4 *g*). The vessel kit was assembled as described above. Distillation was carried out for 18 h at 155 °C. The elevated temperature step was used to thermally decompose the lead hydroxide formed during neutralization (Eq. (1)), converting it to solid lead oxide and releasing any structurally bound hydrogen (Eq. (2)):

(1) PbCl_2_ (s) + 2NaOH (aq) → 2NaCl (s) + Pb(OH)_2_ (aq)

(2) Pb(OH)_2_ (aq) → PbO (s) + H_2_O (l) (> 150 °C)

After distillation, the recovered water distillate was then measured for electrical conductivity and pH analyses. All handling, processing, and disposal of lead-containing materials were performed in accordance with institutional procedures for toxic substances.

### Statistical evaluation

Statistical evaluation was used to assess pre- and post-distillation measurements of water mass, electrical conductivity, stable isotope composition (δ²H and δ¹⁸O), and tritium activity. All statistical comparisons were performed on paired measurements from the same sample before and after distillation.

#### Gravimetric recovery

Gravimetric recovery was calculated for each deionized water sample in the distillation experiments. For this, the ratio of post-distillation water mass recovered in the collection vial to the pre-distillation water mass loaded into the distillation vial is expressed as a percentage. Mass measurements were obtained by weighing vials with caps installed both before and after distillation to minimize evaporative bias. No additional statistical testing was applied to gravimetric recovery beyond calculating percent recovery for each vessel.

#### Conductivity measurements

Electrical conductivity was measured before and after distillation for all saline and alkaline sample types. Conductivity values were reported in µS cm⁻¹. Evaluation of salt removal was based on comparison of pre- and post-distillation conductivities relative to acceptance thresholds for liquid scintillation counting and stable isotope analysis. No transformation of conductivity data was applied.

#### Isotopes comparison pre- and post- distillation

Agreement between pre- and post-distillation isotope measurements (δ²H, δ¹⁸O and ^3^H activities) was evaluated using zeta scores (ζ′), which test whether two values agree within their combined measurement uncertainties. The ζ′-score was calculated per Eq. (3):

(3) $$\zeta =\frac{x_{1}-x_{2}}{\sqrt{u_{1}^{2}+u_{2}^{2}}}$$

where $x_{1}$ is the experimental value, $x_{2}$ is the reference value, and $u_{1}$ and $u_{2}$ are their associated standard uncertainties.

Zeta scores were interpreted according to commonly accepted criteria [14]:

|ζ|≤2:acceptable

2<|ζ|<3:questionable,butOK

|ζ|≥3:unacceptable

In this study, for all samples, the pre-distillation reference value was defined as the mean of triplicate measurements of the initial sample pre-distillation and the experimental value was defined as each individual post-distillation measurement. For all natural abundance stable isotope samples, standard uncertainties of ±1 ‰ for δ²H and ±0.2 ‰ for δ¹⁸O were applied, consistent with common uncertainty values used in data reduction and reporting [15]. For deuterium-enriched samples, an uncertainty of ±35 ‰ was applied [16]. For tritium samples, measurement uncertainties were those reported by the liquid scintillation counting system and included counting statistics and background corrections.

For samples with ^3^H activities below the minimum detectable activity (MDA), ζ′-scores could not be calculated. In these cases, comparison was based on confirmation that both pre- and post-distillation measurements were below the reported MDA.

### Validation data

#### Gravimetric recoveries

Gravimetric recovery was evaluated to assess the completeness of the microdistillation and potential evaporative or mechanical losses during operation of the EvapoClean system. Twelve 12 g aliquots of deionized water (Sample type 1) were distilled simultaneously through all twelve purification ports. Pre- and post-distillation masses were determined by weighing vials with caps in place. Percent recoveries ranged from 99.7 % to 99.9 % across the 12 ports (Table 1).

**Table 1:** Gravimetric recovery of deionized water after overnight (18 h) distillation at 100 °C in the EvapoClean system.

Vial #	Sample Pre-Distillation (g)	Sample post-distillation (g)	Loss (g)	% Loss	% Recovery
1	12.01	11.98	0.03	0.2	99.8
2	12.01	11.99	0.02	0.2	99.8
3	12.00	11.98	0.02	0.2	99.8
4	12.00	11.98	0.02	0.2	99.8
5	12.01	12.00	0.01	0.1	99.9
6	12.01	11.99	0.02	0.2	99.8
7	12.02	11.98	0.04	0.3	99.7
8	12.01	11.99	0.02	0.2	99.8
9	12.01	12.00	0.01	0.1	99.9
10	12.00	11.99	0.01	0.1	99.9
11	12.00	11.99	0.01	0.1	99.9
12	12.02	12.00	0.02	0.2	99.8

#### Removal of salts

Electrical conductivity was measured before and after distillation for NaCl and sea-salt solutions prepared at concentrations of 5, 15, 35, 70, and 100 g L⁻¹ (sample types 2 and 3).

#### Removal of sodium chloride

Pre-distillation conductivities of NaCl solutions ranged from approximately 9000 to ∼130,000 µS cm⁻¹. Post-distillation conductivities were reduced to values between 2 and 37 µS cm⁻¹ (Table 3). These values meet acceptance criteria for downstream liquid scintillation counting and stable isotope analysis.

#### Removal of sea-salt

Pre-distillation conductivities of sea-salt solutions ranged from approximately 6900 to 100,500 µS cm⁻¹. Post-distillation conductivities ranged from 37 to 246 µS cm⁻¹ for most samples, with one 70 g L⁻¹ replicate initially yielding 1015 µS cm⁻¹, indicating salt carryover, which decreased to 46 µS cm⁻¹ following a second redistillation (Table 5). These values also meet acceptance criteria for downstream liquid scintillation counting and stable isotope analysis.

#### Isotope recoveries

For all samples, isotope preservation during microdistillation was evaluated by comparing the isotopic composition of pre- and post- distillation samples. Pre-distillation reference values were defined as the mean of triplicate analyses of the original distilled sample (deionized water or SPK-2) or of the deionized water used to prepare the other test solutions (NaCl, sea-salt and ^2^H-enriched solutions). Post-distillation isotope measurements were compared with the reference values using ζ′-scores calculated from the combined analytical uncertainties, as described in the statistical evaluation section.

#### Isotopic integrity of natural-abundance stable isotopes samples

Stable isotope preservation was evaluated using natural-abundance deionized water and saline test solutions. All δ²H values had a standard uncertainty of ±1 ‰, while pre- δ¹⁸O values averaged a standard uncertainty of ± 0.2 ‰.

Twelve aliquots of deionized water were micro-distilled in each of the twelve purification ports. Pre-distillation δ²H values averaged −78.1 ‰ (VSMOW), while pre-distillation δ¹⁸O values averaged −10.6 ‰. Post-distillation δ²H values ranged between −78.2 and −78.1 ‰, and post-distillation δ¹⁸O values ranged between −10.7 and −10.6 ‰ across all vessels (Table 2).

**Table 2:** Stable isotope (δ²H and δ¹⁸O) composition of deionized water pre- and post-distillation and associated ζ′-scores.

Vial #	δ²H Pre (VSMOW)	SD	δ²H Post (VSMOW)	SD	ζ′	δ¹⁸O Pre (VSMOW)	SD	δ¹⁸O Post (VSMOW)	SD	ζ′
1	−78.1	1.0	−78.1	1.0	−0.02	−10.6	0.2	−10.6	0.2	0.00
2	−78.1	1.0	−78.1	1.0	−0.02	−10.6	0.2	−10.7	0.2	−0.04
3	−78.1	1.0	−78.1	1.0	−0.01	−10.6	0.2	−10.7	0.2	−0.04
4	−78.1	1.0	−78.1	1.0	0.01	−10.6	0.2	−10.6	0.2	0.00
5	−78.1	1.0	−78.2	1.0	−0.08	−10.6	0.2	−10.7	0.2	−0.03
6	−78.1	1.0	−78.1	1.0	−0.01	−10.6	0.2	−10.6	0.2	−0.01
7	−78.1	1.0	−78.2	1.0	−0.07	−10.6	0.2	−10.7	0.2	−0.05
8	−78.1	1.0	−78.1	1.0	−0.01	−10.6	0.2	−10.7	0.2	−0.05
9	−78.1	1.0	−78.1	1.0	−0.02	−10.6	0.2	−10.7	0.2	−0.05
10	−78.1	1.0	−78.2	1.0	−0.06	−10.6	0.2	−10.7	0.2	−0.06
11	−78.1	1.0	−78.2	1.0	−0.04	−10.6	0.2	−10.7	0.2	−0.06
12	−78.1	1.0	−78.2	1.0	−0.05	−10.6	0.2	−10.7	0.2	−0.06

Three or two aliquots of each concentration of each salt solution were distilled individually. Pre-distillation δ²H values of the NaCl reference solution averaged −76.1 ‰ (VSMOW) while pre-distillation δ¹⁸O values averaged −10.5 ‰. Post-distillation δ²H values for all NaCl solutions ranged from −76.2 to −76.1 ‰, and post-distillation δ¹⁸O values ranged from −10.6 to −10.5 ‰ across all aliquots (Table 4).

Pre-distillation δ²H values of the sea-salt reference solution averaged −74.8 ‰ (VSMOW) while pre-distillation δ¹⁸O values averaged −10.3 ‰. Post-distillation δ²H values for all sea-salt solutions ranged from −74.7 to −73.2 ‰, and post-distillation δ¹⁸O values ranged from −10.3 to −9.8 ‰ across all aliquots (Table 6).

Comparison of pre- and post-distillation δ²H and δ¹⁸O values for all natural abundance stable isotope solutions yielded ζ′-scores within the acceptance threshold (|ζ′| ≤ 2) (Tables 2, 4, 6). These results indicate concordance between pre- and post-distillation isotope measurements within stated analytical uncertainties.

#### Isotopic integrity of deuterium-enriched samples

Two synthetic deuterium-enriched waters with target δ²H values of approximately +10,000 ‰ and +20,000 ‰ were micro-distilled in triplicate. For the first solution, the pre-distillation δ²H values averaged 10,469 ‰ (VSMOW), and the post-distillation δ²H values were 10,386 ‰, 10,424 ‰ and 10,502 ‰ for the triplicate analyses. For the second solution, the pre-distillation δ²H values averaged 20,892 ‰ (VSMOW), and the post-distillation δ²H values were 20,794 ‰, 20,856 ‰ and 20,874 ‰ for the triplicate analyses (Table 7).

Agreement between pre- and post-distillation δ²H values was evaluated using ζ′-scores with an assigned uncertainty of ±35 ‰. For each enrichment level, all ζ′-scores were within the acceptable range (|ζ′| ≤ 2 (Table 7).

#### Tritium recovery

Twelve aliquots of the spike solution were micro-distilled in each of the twelve purification ports. The pre-distillation ^3^H activity averaged 35.25 ± 0.79 Bq L⁻¹. Post-distillation ^3^H activities ranged from 33.15 to 36.61 Bq L⁻¹ across the 12 distillation ports (Table 9). Post-distillation activities agreed with pre-distillation values within combined counting uncertainty. All ζ′-scores were within the acceptable range (|ζ′| ≤ 2), indicating quantitative recovery of tritium during microdistillation (Table 8).

#### Memory-effect assessment

##### ^2^H-enriched memory effect assessment

To assess potential carryover, deionized water with natural stable-isotope abundances was distilled in the same vessels after vial cleaning and microdistillation of deuterium-enriched samples. ζ′-scores comparing pre- and post-distillation δ²H and δ¹⁸O values were calculated. All ζ′-scores were within the acceptable range (|ζ′| ≤ 2), indicating concordance with reference values and showing the absence of detectable H isotopic carryover (Table 9).

##### Tritium memory-effect assessment

To evaluate potential carryover, tritium-free water was distilled in the same vessels following the tritium-spiked samples. All measured activities before and after distillation were below the minimum detectable activity (MDA). Because activities were <MDA, ζ′-scores could not be calculated. The absence of detectable tritium in all post-distillation measurements confirmed the absence of carryover (Table 10).

#### PbCl_2_ neutralization and distillation

Electrical conductivity and pH were measured before and after neutralization and distillation of twelve electrolytically enriched samples (sample type 7). Prior to neutralization and distillation, electrical conductivity values for all samples ranged from 270,762 µS cm⁻¹ to 299,232 µS cm⁻¹, while pH values were all measured at 14. After neutralization and distillation, electrical conductivity decreased to 3–24 µS cm⁻¹, and pH decreased to 6–7 for all samples. These values meet the acceptance criteria for LSC analysis of enriched tritium samples.

#### Summary of method performance

• Quantitative recovery of water was achieved during automated microdistillation, with gravimetric recoveries ranging from 99.7 % to 99.9 % across all 12 purification ports (Table 1).
• Distillation reduced the electrical conductivity of a wide range of saline and alkaline samples to levels compatible with downstream liquid scintillation counting and stable isotope analysis for all tested water sample matrices, including sodium chloride and sea-salt solutions (Tables 3 and 5).

**Table 3:** Electrical conductivity (µS cm⁻¹) of NaCl solutions before and after microdistillation in the EvapoClean system.

Vial #	[NaCl] (g L⁻¹)	Electrical Conductivity (pre)	Electrical Conductivity (post)
1	100	127,475	5
2	100	127,475	2
3	100	127,475	3
4	70	95,875	2
5	70	95,875	2
6	70	95,875	2
7	35	54,137	30
8	35	54,137	20
9	35	54,137	37
10	15	25,625	37
11	15	25,625	2
12	15	25,625	3
1	5	8948	2
2	5	8948	2
• Hydrogen and oxygen isotope ratios (*δ*²H and δ¹⁸O) measured before and after microdistillation agreed within stated analytical uncertainties for natural-abundance, saline, and isotopically enriched samples, as evaluated using ζ′-scores (Tables 2, 4, 6, and 7).

**Table 4:** Stable isotope (δ²H and δ¹⁸O) composition of NaCl solutions pre- and post-distillation and associated ζ′-scores.

Vial #	[NaCl] (g L⁻¹)	δ²H Pre (VSMOW)	SD	δ²H Post (VSMOW)	SD	ζ′	δ¹⁸O Pre (VSMOW)	SD	δ¹⁸O Post (VSMOW)	SD	ζ′
1	100	−76.1	1.0	−76.1	1.0	0.01	−10.5	0.2	−10.6	0.2	−0.01
2	100	−76.1	1.0	−76.1	1.0	0.02	−10.5	0.2	−10.5	0.2	0.03
3	100	−76.1	1.0	−76.2	1.0	−0.02	−10.5	0.2	−10.5	0.2	0.02
4	70	−76.1	1.0	−76.2	1.0	−0.03	−10.5	0.2	−10.6	0.2	−0.03
5	70	−76.1	1.0	−76.2	1.0	−0.03	−10.5	0.2	−10.5	0.2	0.02
6	70	−76.1	1.0	−76.2	1.0	−0.03	−10.5	0.2	−10.5	0.2	0.01
7	35	−76.1	1.0	−76.2	1.0	−0.03	−10.5	0.2	−10.6	0.2	−0.03
8	35	−76.1	1.0	−76.2	1.0	−0.04	−10.5	0.2	−10.5	0.2	0.05
9	35	−76.1	1.0	−76.2	1.0	−0.08	−10.5	0.2	−10.5	0.2	0.06
10	15	−76.1	1.0	−76.2	1.0	−0.08	−10.5	0.2	−10.5	0.2	0.03
11	15 15	−76.1	1.0	−76.2	1.0	−0.05	−10.5	0.2	−10.5	0.2	0.04
12		−76.1	1.0	−76.2	1.0	−0.07	−10.5	0.2	−10.5	0.2	0.05
1	5	−76.1	1.0	−76.2	1.0	−0.07	−10.5	0.2	−10.5	0.2	0.04
2	5	−76.1	1.0	−76.2	1.0	−0.08	−10.5	0.2	−10.5	0.2	0.05

**Table 5:** Electrical conductivity (µS cm⁻¹) of sea-salt solutions before and after distillation in the EvapoClean system.

Vial #	[Sea salt] (g L⁻¹)	Electrical Conductivity (pre)	Electrical Conductivity (post)	Conductivity post-2nd distillation
12	100	100,513	148	-
11	100	100,513	200	-
10	100	100,513	246	-
9	70	75,587	1015	46
8	70	75,587	212	-
7	70	75,587	199	-
6	35	43,043	120	-
5	35	43,043	103	-
4	35	43,043	124	-
3	15	20,329	85	-
2	15	20,329	88	-
1	15	20,329	55	-
3	5	6913	55	-
2	5	6913	59	-
1	5	6913	37	-

**Table 6:** Stable isotope (δ²H and δ¹⁸O) composition of sea-salt solutions pre- and post-distillation and associated ζ′-scores.

Vial #	[Sea Saltl] (g L⁻¹)	δ²H Pre (VSMOW)	SD	δ²H Post (VSMOW)	SD	ζ′	δ¹⁸O Pre (VSMOW)	SD	δ¹⁸O Post (VSMOW)	SD	ζ′
12	100	−74.8	1.0	−74.4	1.0	0.27	−10.3	0.2	−10.3	0.2	−0.20
11	100	−74.8	1.0	−74.3	1.0	0.32	−10.3	0.2	−10.3	0.2	−0.14
10	100	−74.8	1.0	−74.4	1.0	0.26	−10.3	0.2	−10.3	0.2	−0.15
9	70	−74.8	1.0	−74.4	1.0	0.27	−10.3	0.2	−10.3	0.2	−0.04
8	70	−74.8	1.0	−74.5	1.0	0.20	−10.3	0.2	−10.3	0.2	−0.14
7	70	−74.8	1.0	−73.2	1.0	1.14	−10.3	0.2	−9.8	0.2	1.80
6	35	−74.8	1.0	−74.5	1.0	0.18	−10.3	0.2	−10.3	0.2	0.05
5	35	−74.8	1.0	−73.5	1.0	0.90	−10.3	0.2	−9.9	0.2	1.46
4	35	−74.8	1.0	−74.5	1.0	0.17	−10.3	0.2	−10.3	0.2	0.06
3	15	−74.8	1.0	−74.5	1.0	0.18	−10.3	0.2	−10.3	0.2	0.11
2	15	−74.8	1.0	−74.5	1.0	0.18	−10.3	0.2	−10.2	0.2	0.15
1	15	−74.8	1.0	−74.5	1.0	0.16	−10.3	0.2	−10.2	0.2	0.18
3	5	−74.8	1.0	−74.6	1.0	0.12	−10.3	0.2	−10.3	0.2	0.09
2	5	−74.8	1.0	−74.7	1.0	0.07	−10.3	0.2	−10.3	0.2	0.08
1	5	−74.8	1.0	−74.7	1.0	0.06	−10.3	0.2	−10.3	0.2	0.05

**Table 7:** Stable H isotope composition ^2^H-deuterium-enriched samples pre- and post-distillation and associated ζ′-scores.

Vial #	Sample	δ²H Pre (VSMOW)	SD	δ²H Post (VSMOW)	SD	ζ′
1	10K	10,469	35	10,502	35	0.66
2	10K	10,469	35	10,424	35	−0.91
3	10K	10,469	35	10,386	35	−1.68
4	20K	20,892	35	20,794	35	−1.97
5	20K	20,892	35	20,856	35	−0.73
6	20K	20,892	35	20,874	35	−0.35
• Distillation of highly deuterium-enriched water did not result in detectable isotopic carryover to subsequent natural-abundance samples, and no memory effects were observed after appropriate cleaning procedures (Table 9).
• Tritium activities measured before and after distillation agreed within combined counting uncertainties, and no detectable tritium carryover was observed in subsequent tritium-free samples (Tables 8 and 10).

**Table 8:** Tritium activity of a tritium-spiked water sample (SPK-2) pre- and post-distillation and associated ζ′-scores.

Vial #	Sample	Pre-Distillation		Post- Distillation		ζ′-Score
		Bq L⁻¹	Error (1σ)	Bq L⁻¹	Error (1σ)	
1	SPK-2	35.25	0.79	33.99	0.78	−1.14
2	SPK-2	35.25	0.79	33.22	0.80	−1.81
3	SPK-2	35.25	0.79	33.22	0.80	−1.81
4	SPK-2	35.25	0.79	35.50	0.83	0.22
5	SPK-2	35.25	0.79	36.13	0.83	0.77
6	SPK-2	35.25	0.79	34.55	0.78	−0.63
7	SPK-2	35.25	0.79	34.65	0.78	−0.53
8	SPK-2	35.25	0.79	34.93	0.82	−0.28
9	SPK-2	35.25	0.79	33.15	0.77	−1.90
10	SPK-2	35.25	0.79	34.12	0.78	−1.01
11	SPK-2	35.25	0.79	36.61	0.88	1.15
12	SPK-2	35.25	0.79	34.46	0.78	−0.71

**Table 9:** Memory-effect test following distillation of deuterium-enriched samples: Stable isotope composition (δ²H and δ¹⁸O) of deionized water pre- and post-distillation and associated ζ′-scores.

Vial #	Sample distilled previously in same vial	δ²H Pre (VSMOW)	SD	δ²H Post (VSMOW)	SD	ζ′	δ¹⁸O Pre (VSMOW)	SD	δ¹⁸O Post (VSMOW)	SD	ζ′
1	10K	−74.8	1.0	−74.5	1.0	0.19	−10.3	0.2	−10.3	0.2	−0.02
2	10K	−74.8	1.0	−74.5	1.0	0.19	−10.3	0.2	−10.3	0.2	0.01
3	10K	−74.8	1.0	−74.5	1.0	0.19	−10.3	0.2	−10.3	0.2	0.00
4	20K	−74.8	1.0	−74.3	1.0	0.35	−10.3	0.2	−10.3	0.2	0.00
5	20K	−74.8	1.0	−74.3	1.0	0.34	−10.3	0.2	−10.3	0.2	−0.02
6	20K	−74.8	1.0	−74.3	1.0	0.33	−10.3	0.2	−10.3	0.2	0.01

**Table 10:** Memory-effect test following a run of tritium-spiked distillation: Tritium activity of tritium-free water pre- and post-distillation and associated ζ′-scores.

Vial #	Sample	Pre-Distillation			Post- Distillation		
		Bq L⁻¹	Error (1σ)	DL	Bq L⁻¹	Error (1σ)	DL
1	^3^H-free water	<1.11		*	<1.16		*
2	^3^H-free water	<1.11		*	<1.11		*
3	^3^H-free water	<1.11		*	<1.11		*
4	^3^H-free water	<1.11		*	<1.11		*
5	^3^H-free water	<1.11		*	<1.11		*
6	^3^H-free water	<1.11		*	<1.16		*
7	^3^H-free water	<1.11		*	<1.11		*
8	^3^H-free water	<1.11		*	<1.16		*
9	^3^H-free water	<1.11		*	<1.11		*
10	^3^H-free water	<1.11		*	<1.16		*
11	^3^H-free water	<1.11		*	<1.16		*
12	^3^H-free water	<1.11		*	<1.16		*

*Measurement < MDA (Minimum Detection Activity.
• Combined neutralization and distillation resulted in low electrical conductivity and pH suitable for LSC counting and stable isotope analyses (Table 11).

**Table 11:** Electrical conductivity (µS cm⁻¹) and pH of enriched tritium solutions (post-enrichment) Pre- and Post- PbCl_2_ neutralisation + distillation in the EvapoClean system at 155C.

Vial #	Sample ID	Electrical Conductivity Pre-	pH Pre-	Electrical Conductivity Post-	pH Post-
1	1	292,239	14	4	6–7
2	2	294,393	14	3	6–7
3	3	298,804	14	4	6–7
4	4	273,418	14	8	6–7
5	5	299,232	14	3	6–7
6	6	270,762	14	4	6–7
7	7	274,041	14	3	6–7
8	8	271,890	14	6	6–7
9	9	272,357	14	6	6–7
10	10	294,450	14	17	6–7
11	11	271,533	14	24	6–7
12	12	283,399	14	9	6–7
• Overall, performance metrics were comparable to or exceeded typical recovery or EC values reported for conventional open-system or glassware-based distillation methods.

### Limitations

Despite its advantages, some limitations should be considered. The microdistillation unit is limited to processing twelve samples per run; applications requiring larger batch sizes would therefore necessitate the use of multiple units. In addition, unlike classical glass distillation systems, the distillation vials are enclosed within the purification ports during operation, preventing direct observation of boiling behaviour and distillation progress.

## Related research article

None.

## Ethics statements

Not applicable.

## CRediT authorship contribution statement

**Daisy Butera:** Methodology, Data Analysis, Validity tests, Writing – original draft. **Leonard I. Wassenaar:** Conceptualization, Methodology, Editing, Supervision. **Anthony Lapp:** Methodology, Editing. **Paul Middlestead:** Stable isotope analyses, editing. **Oliver Warr:** Supervision, Editing.

## Declaration of competing interest

The authors declare that they have no known competing financial interests or personal relationships that could have appeared to influence the work reported in this paper.

## Data Availability

Data will be made available on request.
